# The Role of TAR DNA Binding Protein 43 (TDP-43) as a CandiDate Biomarker of Amyotrophic Lateral Sclerosis: A Systematic Review and Meta-Analysis

**DOI:** 10.3390/diagnostics13030416

**Published:** 2023-01-23

**Authors:** Caterina Maria Gambino, Anna Maria Ciaccio, Bruna Lo Sasso, Rosaria Vincenza Giglio, Matteo Vidali, Luisa Agnello, Marcello Ciaccio

**Affiliations:** 1 Department of Biomedicine, Neurosciences and Advanced Diagnostics, Clinical Molecular Medicine and Clinical Laboratory Medicine, Institute of Clinical Biochemistry, University of Palermo, 90127 Palermo, Italy; 2 Department of Laboratory Medicine, University Hospital “P. Giaccone”, 90127 Palermo, Italy; 3 Internal Medicine and Medical Specialties “G. D’Alessandro”, Department of Health Promotion, Maternal and Infant Care, University of Palermo, 90127 Palermo, Italy; 4 Foundation IRCCS Ca’ Granda Ospedale Maggiore Policlinico, 20122 Milan, Italy

**Keywords:** TDP-43, biomarker, ALS, diagnosis

## Abstract

Background: TAR DNA-binding protein 43 (TDP-43) aggregation in neuronal cells is recognized as a hallmark of amyotrophic lateral sclerosis (ALS). Although the literature strongly supports the pathogenetic role of TDP-43 in ALS pathogenesis, the role of TDP-43 as a biomarker of ALS is controversial. We performed a systematic review and meta-analysis to assess the diagnostic performance of TDP-43 for ALS. Methods: Relevant publications were identified by a systematic literature search on PubMed and Web of Science from their inception to 8 April 2022. Results: Seven studies, including 472 individuals, of whom 254 had ALS according to the Revised Amyotrophic Lateral Sclerosis Functional Rating Scale, met the inclusion criteria for our meta-analysis. According to the random-effects model, CSF TDP-43 levels are higher in ALS patients compared with control groups. Conclusions: CSF TDP-43 could represent a biomarker of ALS, but further studies are mandatory before drawing conclusions.

## 1. Introduction

Amyotrophic lateral sclerosis (ALS) is a neurodegenerative disease characterized by the progressive loss of both upper and lower motor neurons (UMN and LMN, respectively). On average, patients with ALS suffer from progressive muscle weakness leading to respiratory failure and death within three to five years [1,2]. According to the onset, ALS can be classified as bulbar or spinal. Moreover, up to 50% of patients also develop cognitive and/or behavioral impairment, including frontotemporal lobar degeneration, affecting 13% of ALS cases [3,4,5].

Two forms of the disease are known, sporadic, occurring in 90% of ALS cases, and familiar, due to mutations in more than 20 genes, including hexanucleotide expansions in chromosome 9 open reading frame 72, superoxide dismutase 1, TAR DNA-binding protein 43, fused in sarcoma and TANK-binding kinase 1 [6,7]. The familial form differs clinically from the sporadic form in the younger age of onset and slightly more rapid disease progression.

At present, the diagnosis of ALS is primarily clinical and represents a challenge for clinicians. Specifically, there is no marker or procedure to establish the diagnosis of ALS reliably. The diagnosis is reached through several clinical examinations and diagnostic tests to rule out diseases mimicking ALS. According to El Escorial/Airlie House Criteria [8], the ALS diagnosis is based on three factors: (1) evidence of LMN degeneration; (2) evidence of UMN disease; and (3) the presence of either (UMN and LMN) in more than one body district. Additionally, the course must be progressive with no possible alternative diagnosis. The great heterogeneity of the disease and the non-specific nature of the initial symptoms lead to a significant diagnostic delay. Additionally, since ALS is a rare disease, the clinical diagnostic suspicion is late, and the delay from symptom onset to diagnosis is substantial [2].

With the number of cases expected to rise to nearly 400,000 worldwide by 2040 [9], ALS represents a substantial unmet medical need. Thus, there is intense research for identifying biomarkers to assist clinicians in the appropriate management of ALS patients, from its early detection to guiding and monitoring therapy [10,11].

The ideal biomarker of ALS should reflect the pathophysiological mechanism of the disease. A characteristic feature of degenerating motor neurons in ALS patients is the presence of the cytoplasmic aggregation of TAR DNA-binding protein 43 (TDP-43). TDP-43 is a protein of 414 amino acids, encoded by the TARDBP gene, belonging to the family of heterogeneous nuclear ribonucleoproteins, a group of proteins involved in RNA processing [12].

It is abundantly expressed in systemic organs, such as the central nervous system, pancreas, and spleen. Physiologically, it is localized in the nucleus but under stress conditions, a hyperphosphorylated, ubiquitinated, and cleaved form of TDP-43 aggregates in the cytoplasm, causing axonal swelling and impairing mobility [13,14,15]. Recent evidence reporting TARDBP mutations in a subset of familial ALS associated with pathological TDP-43 accumulation confirmed the pivotal role of aberrant TDP-43 in neurodegeneration [16]. Additionally, neuropathologic evidence from postmortem studies showed the presence of TDP-43 in the ALS brain. The pathological effect of TDP-43 could result from two opposite mechanisms, a gain-of-neurotoxicity of aggregated TDP-43 or a loss-of-function of nuclear TDP-43. Experimental studies showed that the overexpression and aggregation of TDP-43 are associated with neuronal death and axonal dysfunction, highlighting the neurotoxic effect of TDP-43. By contrast, neuronal loss was observed in transgenic mice with impaired TDP-43 nuclear expression and sparse cytoplastic inclusion. Additionally, knock-out mice displayed neuronal dysfunction and synaptic alterations [17,18]. Altogether the literature strongly supports the pathogenetic role of TDP-43 in ALS pathogenesis, the role of TDP-43 as a biomarker of ALS is controversial.

We performed a systematic review and comprehensive meta-analysis to investigate whether TDP-43 levels in cerebrospinal fluid (CSF) could be a reliable biomarker of ALS.

## 2. Materials and Methods

We followed the Preferred Reporting Items for Systematic Reviews and Meta-Analysis (PRISMA) Guidelines 2020 [19]. All studies investigating the diagnostic efficacy of TDP-43 for ALS were searched for inclusion.

### 2.1. Literature Search Strategy

Two reviewers (CMG and AMC) systematically and independently performed a comprehensive electronic search of PubMed and Web of Science. The following medical subject heading (MeSH) terms “TAR DNA-binding protein 43” OR “TDP-43” AND “Amyotrophic Lateral Sclerosis” OR “ALS” AND “CSF” OR “plasma” were used to search articles. No publication date restriction was applied, and the end-date of our search was 08 April 2022.

### 2.2. Study Selection

The inclusion criteria were:

- the manuscript describes a retrospective and prospective study design;
- it was written in the English language;
- the study must provide a full description of the method used to measure TDP-43 levels;
- the study must use TDP-43 as a biomarker in the blood or CSF to differentiate ALS patients from a control group;
- the study must use a quantitative method for TDP-43 detection;
- the study must provide sufficient data to calculate the outcome.

Exclusion criteria were:

- evaluation of only the prognostic role of TDP-43;
- studies without healthy controls or without disease control;
- letters, case reports, animal studies, reviews, and meta-analyses;
- languages other than English;
- qualitative method for TDP-43 detection;
- full text not found.

### 2.3. Data Collection

Two authors (CMG and AMC) independently collected data referring to the study and patient characteristics. The extracted information from each study included the first author’s name, the year of publication, country, study design, clinical setting, study population, sample, the method used, and outcome data (area under the curve (AUC), sensitivity, specificity, and cut-off).

### 2.4. Statistical Analysis

For each study, the standardized between-group mean difference (SMD), calculated as the difference in means between ALS and controls divided by the pooled standard deviation (SD) of both groups, was used as effect size [20]. A small-sample bias correction was applied to these standardized mean differences, obtaining the Hedges’ g effect sizes [21]. When only median and interquartile ranges were available, we used mean = median and SD = IQR/1.35, according to the Cochrane handbook. When the standard error of the mean (SEM) was only available, we used SD = SEM$\sqrt{n}$, where *n* was the sample size.

A random-effects model was used to pool effect sizes. The restricted maximum likelihood estimator [22] was used to calculate the heterogeneity variance, tau^2. Heterogeneity was also quantified by the inconsistency (I^2) index [23]. The Knapp–Hartung adjustments [24] were used to calculate the confidence interval around the pooled effect. Publication bias was tested by Egger’s and Begg’s tests and by funnel plot. Meta-analytical summaries were represented by Forest plots. Meta-analysis was performed with R Language v. 4.2.1 (R Foundation for Statistical Computing, Vienna, Austria) and RStudio IDE v.2022.07.2 (RStudio, PBC, Boston, MA) with the R package “meta”.

## 3. Results

### 3.1. Study Selection

**Figure 1:**
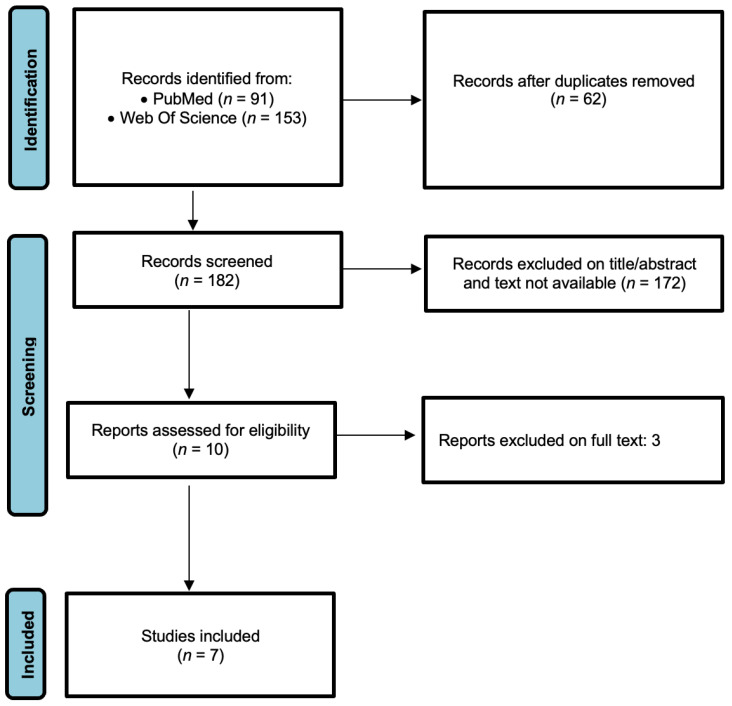
PRISMA 2020 study-selection flow diagram.

The study selection process is schematically presented in the PRISMA flow diagram (Figure 1). A total of 244 articles (91 from PubMed and 153 from Web of Science) were obtained. After the removal of 62 duplicates, 182 studies were retrieved. After screening the title and abstracts, 172 studies were excluded because they were literature reviews, case reports, abstracts, experimental studies on animals, performed only on ALS patients, did not measure TDP-43 by quantitative methods, or did not evaluate the diagnostic accuracy of TDP-43 for ALS. The full text of 10 studies was further evaluated. Finally, a total of seven studies were included in the meta-analysis [25,26,27,28,29,30,31].

### 3.2. Study Characteristics and Quality Assessment

The main characteristics of the studies included in the meta-analysis are described in Table 1.

We used the QUADAS-2 (Quality Assessment of Diagnostic Accuracy Studies) criteria to assess the quality of seven papers selected for systematic review [32]. This tool comprises four domains that assess the risk of bias, covering patient selection, index test, reference standard, and flow and timing. The assessment was performed independently by two authors (CMG and AMC). If there was disagreement, the third author, MV, was consulted. The results of the quality assessment of selected papers are summarized in Table 2.

### 3.3. Diagnostic Accuracy of TDP-43 for ALS

The restricted maximum likelihood method estimated a between-study heterogeneity variance of tau^2 = 0.27 (95%CI 0.06–1.54). The I^2 index was 79.2% (95%CI 57.4%-89.9%), suggesting substantial heterogeneity.

**Figure 2:**
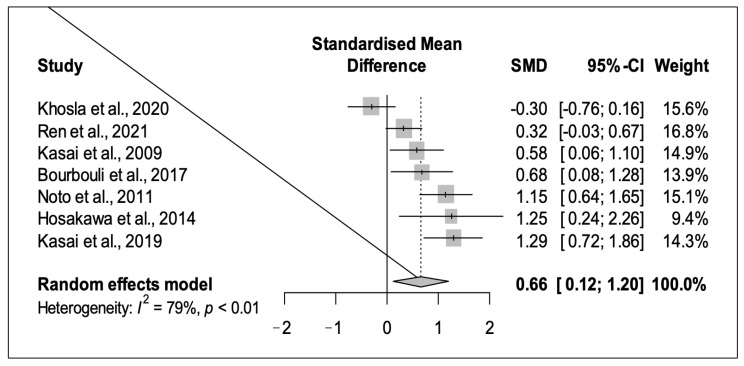
Forest plot of the pooled SMD (diamond) under the random effects model. See refs. [25,26,27,28,29,30,31].

According to the random effects model, the pooled effect was g = 0.66, with the 95%CI ranging from 0.12 to 1.20. The effect was statistically significant (*p* = 0.024), indicating that TDP-43 in CSF was higher in ALS patients than in controls. Figure 2 reports the SMDs and the pooled meta-analytical summary (Figure 2).

No publication bias was detected by either Egger’s test (*p* = 0.224) or Begg’s test (*p* = 0.177).

## 4. Discussion

Since its discovery fifteen years ago, several pieces of evidence from clinical and experimental studies have revealed the pathogenic role of TDP-43 in ALS. It is now widely known that it binds to UG-rich repeats of target RNAs, and it has a pivotal role in regulating mRNA transcription, splicing, transport, and stability. Under physiological conditions, TDP-43 is expressed in the nucleus and can shuttle between nucleus and cytoplasm [33]. However, under pathological conditions, it can translocate to the cytoplasm where forms amyloid fibrils and inclusion bodies leading to neuronal cell death (we suggest reading the review by Chen et al. for more details [34]). TDP-43 cytoplasmatic inclusions are detected in up to 97% of ALS patients and, thus, it is now regarded as a pathological hallmark of ALS [35]. Recently, in vitro studies suggested that neurodegeneration due to TDP-43 could be the result not only of cytoplasmatic inclusions but also of other processes related to the impairment of its physiological functions, i.e., RNA instability [36,37,38].

In this systematic review and meta-analysis, we evaluated the usefulness of CSF TDP-43 as a biomarker of ALS by analyzing results from seven studies, including a total of 472 individuals, 254 patients with ALS and 198 controls. We found that CSF TDP-43 was significantly increased in ALS patients compared with controls. The difference among sample means was standardized by the pooled standard deviation, since standardization makes it much easier to evaluate the magnitude of the mean difference. Following the conventions by Cohen [20], the pooled SMD found of 0.66 is considered a moderate-to-large effect. Thus, CSF TDP-43 could represent a biomarker of ALS. Only a few authors have evaluated the diagnostic performance of TDP-43 in ALS. Bourbouli et al. reported good diagnostic performance of TDP-43 for diagnosing ALS with an AUC of 0.75 (0.60–0.86), and a sensitivity and specificity of 72% and 77%, respectively [28]. Interestingly, the combination by a mathematical formula of TDP-43 with two biomarkers of neurodegeneration, namely total tau and tau phosphorylated at threonine 181, reached optimal sensitivity and specificity (>90%) with an AUC of 0.97 (0.82–0.99). Additionally, Kasai et al. found a good diagnostic accuracy of CSF TDP-43 for diagnosing ALS with an AUC of 0.802 [27]. The combination of neurofilament light and TDP-43 improved the diagnostic accuracy with an AUC of 0.9493. Noto et al. found that CSF TDP-43 discriminated ALS from other neurological diseases with a specificity of 96% and a sensitivity of 59.3%; the AUC was not stated [30].

The research in the field of ALS biomarkers is hampered by several factors, including the rarity of the disease, the delayed diagnosis, and the high rate of misdiagnosis. While improvements in biotechnology have led to significant progress for several neurological diseases, such as Alzheimer’s disease [39,40,41], the biomarker discovery of ALS is strongly slowed down. In the last decades, TDP-43 has gained attention, and preliminary data are promising. However, much effort is needed before introducing it in clinical practice. A significant limitation of most studies is the small sample size.

Currently, studies on CSF TDP-43 in ALS are highly heterogenous in terms of sample size, the analytical assay used for measuring the biomarker, and inclusion and exclusion criteria for patients and, especially, for controls. Indeed, some authors included healthy individuals [25,28], while most authors included patients with neurological diseases that should not influence TDP-43 levels, such as multiple sclerosis [27,29,30,31]. All these factors contributed to the heterogeneity among studies we observed in this meta-analysis. Accordingly, we used a random-effects model to pool effect sizes due to high heterogeneity. Moreover, a correction for small-sample bias was applied.

A few studies evaluated the concentration of TDP-43 in plasma, achieving inconsistent results. This could be partially explained by the analytical methods used. Indeed, most authors measured TDP-43 by ELISA, and the commercially available kits have insufficient sensitivity to detect circulating TDP-43. The Simoa analyzer has better analytical performance with a 100- to 1000- higher sensitivity than ELISA. Kasai et al., by measuring TDP-43 using Simoa analyzer in a cohort of 29 ALS patients and 29 age-matched controls, found increased levels of the biomarker in both the CSF and plasma of ALS patients [27]. Although the study population was very small, the cohorts were very well-characterized. Similarly, Ren et al. found higher levels of TDP-43 both in the CSF and plasma of ALS patients than in healthy controls [25]. It is noteworthy that they measured the biomarker’s levels by sandwich ELISA. The most interesting finding is that plasma TDP-43 displayed a significantly better accuracy for diagnosing ALS than that of CSF (AUC = 0.924 and 0.588 for plasma and CSF TDP-43, respectively). The authors provide some possible explanations for this discrepancy. Briefly, one concerns preanalytical factors and especially the material of the tubes used for collecting and storing samples. Another is related to the different stability of TDP-43 in CSF and blood due to the presence of protease in the CSF. However, the study had several limitations.

In conclusion, in this meta-analysis we evaluated only CSF TDP-43 and not plasma, since the number of studies was too low. Overall, the findings of this meta-analysis encourage further studies to validate the possible role of TDP-43 as a biomarker of ALS.

Some limitations of this study should be cited. First, we performed the statistical analysis on a small number of studies and each study included a small sample size. Additionally, different analytical methods and, consequently, different concentrations were reported in the included studies.

## Figures and Tables

**Table 1:** Characteristics of the studies included in the meta-analysis.

Author, Year	Study Setting	Study Population		Mean Age (Years)		Male/Female		Disease Duration (Months)	ALSFRS-R	Sample	Analytical Method
		Total	ALS	ALS	Controls	ALS	Controls				
Ren et al., 2021 [25]	China	128	69	51.46 (34–69)	51.76 (23–76)	46/23	37/22	19.59 (3–60)	37.91 (18.47)	Plasma and CSF	ELISA
Khosla et al., 2020 [26]	India	86	54	48.01 ± 12.24	38.12 ± 16.43	43/10	28/4	19.34	34.37 ± 6.17	CSF	ELISA
Kasai et al., 2019 [27]	Japan	53	29	65.41 ± 12.34	66.40 ± 9.2	18/11	19/10	NA	NA	Plasma and CSF	Simoa assay
Bourbouli et al., 2017 [28]	Greece	49	32	61.4 ± 9.47	59.7 ± 10.2	17/15	30/21	15.24 ± 11.4	43 (42–46)	CSF	ELISA
Hosakawa et al., 2014 [29]	Japan	20	13	58.9 ± 15.65	48.8 ± 21.58	7/6	5/2	21 ± 26.7	NA	CSF	ELISA
Noto et al., 2011 [30]	Japan	77	27	NA	NA	NA	NA	NA	NA	CSF	ELISA
Kasai et al., 2009 [31]	Japan	59	30	NA	NA	19/11	NA	14.9 ± 10.6	NA	CSF	ELISA

Total: total sample size, ALS: amyotrophic lateral sclerosis; ALSFRS-R: revised amyotrophic lateral sclerosis functional rating scale; CSF: cerebrospinal fluid; NA, information not available; ELISA: enzyme linked immunosorbent assay.

**Table 2:** Quality assessment of the studies included in the meta-analysis.

Author, Year	Representative Spectrum	Acceptable Reference Standard	Acceptable Delay between Tests	Partial Verification Avoided	Differential Verification Avoided	Incorporation Avoided	Reference Standard Results Blinded	Index Test Results Blinded	Uninterpretable Results Reported	Withdrawals Explained	Sponsoring Precluded
Ren et al., 2021	YES	YES	YES	YES	YES	YES	YES	YES	NO	YES	UNCLEAR
Khosla et al., 2020	YES	YES	YES	YES	YES	YES	NO	UNCLEAR	NO	YES	UNCLEAR
Kasai et al., 2019	YES	UNCLEAR	UNCLEAR	YES	YES	YES	NO	NO	NO	UNCLEAR	YES
Bourbouli et al., 2017	YES	YES	YES	YES	YES	YES	YES	YES	NO	YES	YES
Hosakawa et al., 2014	YES	YES	YES	YES	YES	YES	NO	NO	NO	YES	YES
Verstraete et al., 2012	YES	YES	YES	YES	YES	YES	NO	NO	NO	YES	YES
Noto et al., 2011	YES	YES	YES	YES	YES	YES	NO	NO	NO	YES	YES

Green, low risk of bias; Yellow, medium risk of bias; Red, high risk of bias.

## Data Availability

Not applicable.
